# The effect of locally delivered cisplatin is dependent on an intact immune function in an experimental glioma model

**DOI:** 10.1038/s41598-019-42001-7

**Published:** 2019-04-04

**Authors:** Julio Enríquez Pérez, Sara Fritzell, Jan Kopecky, Edward Visse, Anna Darabi, Peter Siesjö

**Affiliations:** 1 Glioma Immunotherapy Group, Division of Neurosurgery, Department of Clinical Sciences, Lund University, Lund, Sweden; 2 Division of Neurosurgery, Department of Clinical Sciences, Skåne University Hospital, Lund, Sweden

## Abstract

Several chemotherapeutic drugs are now considered to exert anti-tumour effects, by inducing an immune-promoting inflammatory response. Cisplatin is a potent chemotherapeutic agent used in standard medulloblastoma but not glioblastoma protocols. There is no clear explanation for the differences in clinical efficacy of cisplatin between medulloblastomas and glioblastomas, despite the fact that cisplatin is effective *in vitro* against the latter. Systemic toxicity is often dose limiting but could tentatively be reduced by intratumoral administration. We found that intratumoral cisplatin can cure GL261 glioma-bearing C57BL/6 mice and this effect was abolished in GL261-bearing NOD-scid *IL2rγ*^*null*^ (NSG) mice. Contrary to previous results with intratumoral temozolomide cisplatin had no additive or synergistic effect with whole cell either GL261 wild-type or GM-CSF-transfected GL261 cells whole cell vaccine-based immunotherapy. While whole tumour cell immunizations increased CD8^+^ T-cells and decreased F4/80^+^ macrophages intratumorally, cisplatin had no effect on these cell populations. Taken together, our results demonstrate that intratumoral cisplatin treatment was effective with a narrow therapeutic window and may be an efficient approach for glioma or other brain tumour treatment.

## Introduction

Platinum-based drugs, such as cisplatin (*cis*-diamminedichloroplatinum-II), are among the most widely used chemotherapeutic agents and have shown efficacy against various solid neoplasms outside the central nervous system as testicular, ovarian, breast, colorectal, lung, head and neck tumours^1^. Although cisplatin has been shown to have cytotoxic effects on human glioblastoma cells *in vitro*^2,3^, the response in clinical treatment is weak and has not improved the overall survival of patients with brain tumours as a single agent^4,5^ or in combinations with radiotherapy^6,7^ and chemotherapeutic drugs such as carmustine (BCNU)^8–10^ and temozolomide (TMZ)^11^. Cisplatin is an integral part of standard treatment of medulloblastomas^12,13^ but the systemic toxicity of cisplatin, including nephrotoxicity, haematological toxicity, peripheral neurotoxicity and ototoxicity, is a restricting factor^6,7,14,15^ and less than 20% of patients treated for medulloblastoma receive the stipulated dose^13^. There is no clear explanation to the differences in clinical efficacy of cisplatin between medulloblastomas and glioblastomas.

Systemically delivered cisplatin penetrates poorly into normal brain tissue due to the blood-brain barrier (BBB) with less than 5% of the plasma concentration detected in the brain after intravenous delivery^16^. However, the neo-vasculature in tumours is more permeable than the intact BBB, and therapeutic cisplatin levels have been detected in primary and secondary brain tumours and to a lesser extent in the oedematous brain adjacent to tumour after systemic delivery^17,18^. Convection enhanced delivery (CED) is a technique that use positive pressure to deliver a cytostatic drug intratumorally, aiming to increase the intratumoral concentrations while decreasing the systemic toxicity^19^. Cisplatin is described as an alkylating-like drug because of its ability to crosslink with the purine bases, resulting in distortion of the DNA structure by bending and unwinding of the double helix. This interferes with the DNA repair mechanisms, causing DNA damage and subsequently apoptosis in cancer cells^1,12^.

Several chemotherapeutic drugs and radiotherapy are now considered to exert anti-tumour properties not only by direct tumour cell killing but also by inducing an immune-promoting inflammatory response, which depending of the immunogenicity of the dying tumour cell^20,21^. Among the immune-promoting effects, induction of immunogenic cell death (ICD) has gained most attention. The ICD process comprises secretion, release or exposure of damage-associated molecular patterns (DAMPs) such as calreticulin, ATP and HMGB1^22–24^ which initiate an effective priming of an immune response against antigens released from dying cells^25^. Cisplatin has been described as a non-*bona fide* or partial ICD inducer; it induces ATP secretion and translocation of HMGB1 from the cell nucleus to the cytoplasm, but its inability to induce endoplasmic reticulum (ER) stress prevents calreticulin translocation from the ER to the outer surface of the plasma membrane^25,26^. Cisplatin may also modulate anti-tumour immunity through other mechanisms, such as improving the recruitment and proliferation of immune effector cells, augmenting their lytic activity, up-regulating MHC-I and downregulating immunosuppression in the tumour microenvironment^21^.

Given the desolate prognosis, there is an urgent need for development of novel therapies against glioblastomas. We have previously reported that glioma-bearing mice could be cured by peripheral whole cell immunizations using granulocyte macrophage colony-stimulating factor (GM-CSF)-transduced tumour cells in the GL261 mouse glioma model, and that the therapeutic effect was mediated by both CD4^+^ and CD8^+^ T cells^27,28^. Additionally CED of TMZ in the same model had a curative and immune dependent effect that synergized with whole cell immunizations^29^. The immune modulating effects of cisplatin suggests a potential therapeutic gain by combining immunotherapy with cisplatin-based chemotherapy. Therefore, in the present study we aimed to investigate the therapeutic efficacy of intratumorally delivered cisplatin as a single agent or in combination with whole cell vaccine-based immunotherapy as peripheral immunizations using GL261 wild type (GL-wt) and GM-CSF-producing GL261 cells (GL-GM) in the mouse glioma GL261 model.

## Results

### GL261 cells are sensitive to cisplatin exposure *in vitro*

**Figure 1:**
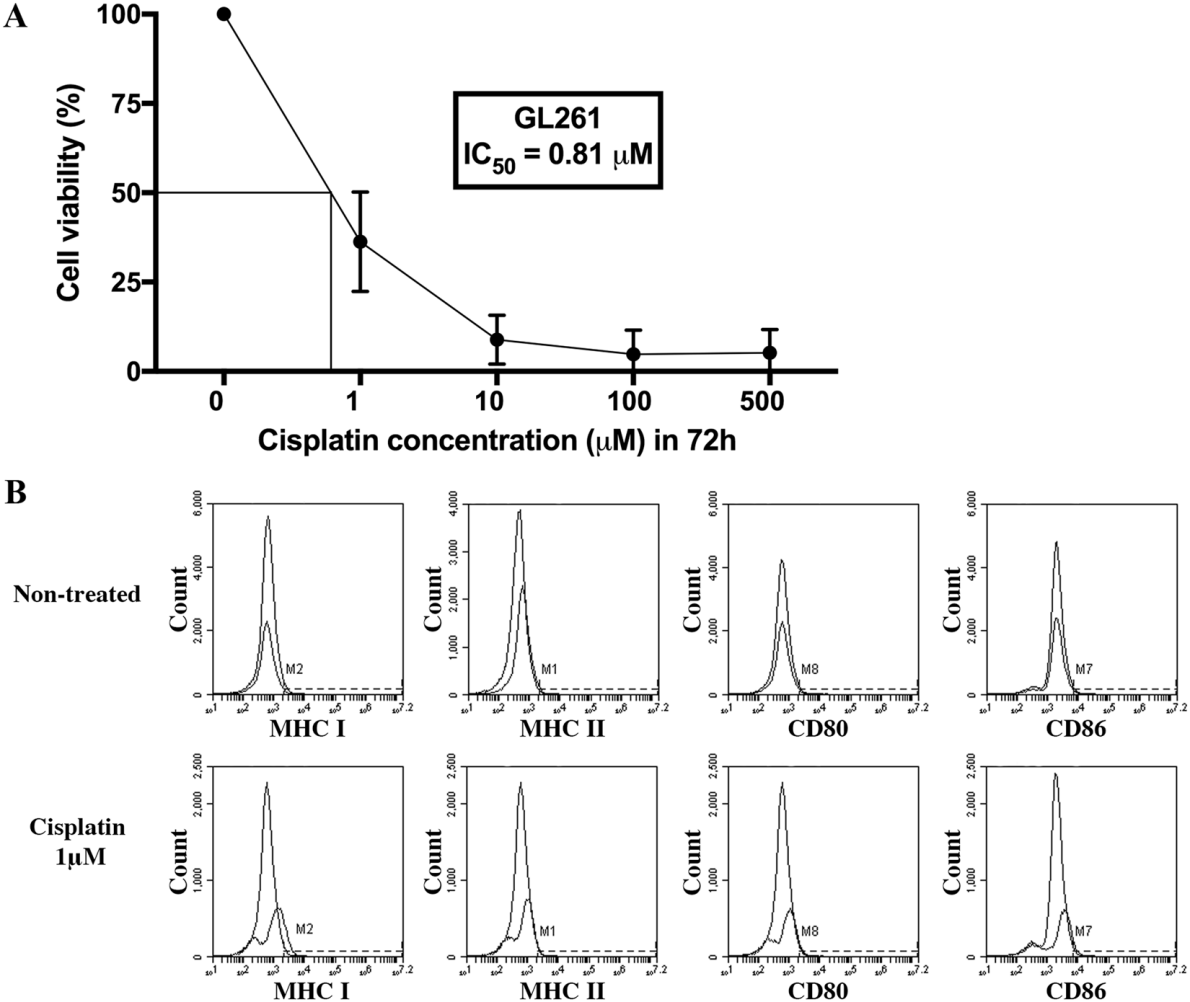
Cell viability and expression of MHC class I and II, CD80 and CD86 on GL261 cells following cisplatin exposure *in vitro*. GL261 cells were daily exposed to different doses of cisplatin for 3 days (72 hours). The following day, cells were analysed using flow cytometry for (**A**) viability using 7AAD staining (percentage of treated viable cells/non-treated viable cells, where the viability of the non-treated cells was regarded as 100%, mean values out of triplicates) or (**B**). No changes in expression of MHC class I, MHC class II, CD80 and CD86 following cisplatin exposure (1 μM), Histograms from 1 out of 3 experiments are presented.

To determine the cytotoxic effects of cisplatin on the GL261 cell line, cells were exposed to a range of cisplatin concentrations *in vitro* for 3 days and the viability of the cells was analysed the following day by 7AAD staining using flow cytometry. Cisplatin was active against the cell line *in vitro* with a median IC_50_ of 0.81 μM and cytotoxicity increased with increasing doses of cisplatin (Fig. 1A, mean of triplicate samples). The cell viability of GL261 was 37% at 1 μM and decreased to 6% at 10 μM. At 100–500 μM the cell viability of the GL261 cells was less than 5%.

### MHC expression of GL261 cells increases following cisplatin exposure *in vitro*

We have earlier shown that interferon-γ exposure, TMZ and irradiation upregulate both MHC class I and II on GL261 cells *in vitro*^27,29^. Since MHC expression affects the immunogenicity of cells, we investigated the expression of MHC, as well as other surface proteins linked to antigen presentation, on the cell surface after cisplatin exposure *in vitro* for 3 days. Exposed GL261 cells were stained with anti-MHC class I, anti-MHC class II, anti-CD80 and anti-CD86 antibodies and analysed by flow cytometry. Non-treated GL261 cells had low expression of MHC class I, MHC class II, CD80 and CD86, less than 1% were positive (Fig. 1B). We found a trend of increased MHC class I expression following cisplatin (1 μM) exposure (Fig. 1B). The percentage of cells expressing MHC class II, CD80 and CD86 did not change following cisplatin exposure (Fig. 1B).

### CED of cisplatin induces cure in the GL261 model

**Figure 2:**
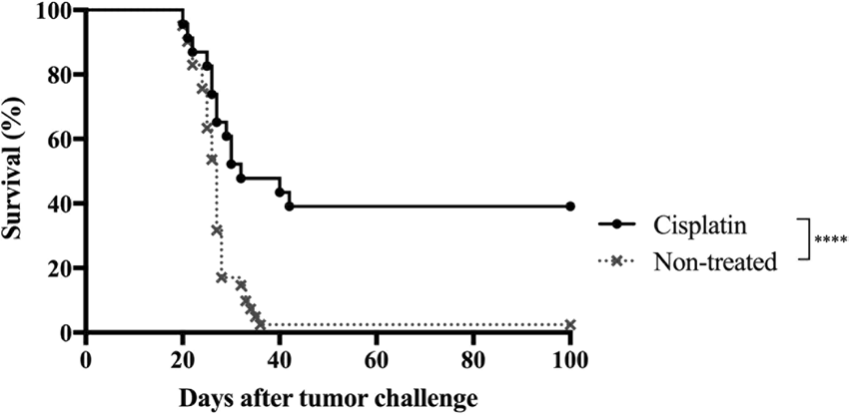
Effect of CED of cisplatin on survival of GL261 tumour-bearing C57BL/6 mice. Kaplan-Meier survival curves of GL261-bearing mice receiving CED of cisplatin using a mini-osmotic pump/brain infusion kit at days 7–9, 0.01 μg/μl/h (*n* = 23) and non-treated mice (*n* = 41). The survival rate of treated mice was 39%; 1 mouse was excluded from the graph due to signs of toxicity of cisplatin on day 14. Non-treated vs. cisplatin (****p < 0.0002), log rank test. Survival was monitored for 100 days.

Next, we investigated the efficacy and toxicity of CED of cisplatin. C57BL/6 mice carrying intracranial GL261 tumours were treated with different doses of cisplatin using mini-osmotic pumps. The highest dose of cisplatin (0.9 μg/μl/h, total dose 64.8 μg/kg/day) was lethally toxic to 33% (2 of 6 mice) of the treated mice but could cure 25% (1 of 4 mice) of the remaining subjects. Therefore, a lower dose was tested (0.1 μg/μl/h, total dose 7.2 μg/kg/day), but it was also toxic to 60% (3 of 5 mice). The toxicity was reduced by the lowest dose (0.01 μg/μl/h, total dose 12 μg/kg/day) to 4.1% (1 of 24 mice) (Table 1). Mice treated with the lowest dose of cisplatin had a survival rate of 39% (9 of 22 mice) which was significantly different (*p* < 0.0002) when compared with non-treated mice (Fig. 2).

**Table 1:** Toxicity and survival of CED of cisplatin on day 7–9 of GL261-bearing mice.

Strain	Dose	Total dose	Toxicity		Day of toxicity	Survival	
	(μg/μl/h)	(μg)	(n)	(%)		(n)	(%)
C57BL/6	0.9	64.8	2 (6)	33.3	13^a^	1 (4)	25.0
	0.1	7.2	3 (5)	60.0	13–15^a^	0 (2)	0.0
	0.01	0.72	1 (24)	4.16	14^a^	9 (23)	39.1
	0 (NaCl)	0	0 (6)	0.0	—	0 (6)	0.0
NSG	0.01	0,72	1 (6)	16.6	17^a^	0 (6)	0.0
	0 (NaCl)	0	0 (6)	0.0	—	0 (6)	0.0

^a^Toxicity signs: seizures, jerky or slow movements and brain swelling.

### CED of cisplatin fails to cure immunocompromised mice with intracranial tumours

**Figure 3:**
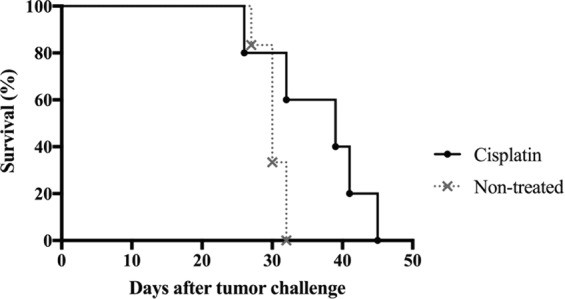
Effect of CED of cisplatin on survival of GL261 tumour-bearing NSG mice. Kaplan-Meier survival curves of GL261-bearing mice receiving CED of cisplatin using a mini-osmotic pump/brain infusion kit at days 7–9, 0.01 μg/μl/h (*n* = 6) or non-treated mice (*n* = 6); 1 mouse was excluded from the graph due to signs of toxicity of cisplatin on day 17. No significant difference was found between treated and non-treated mice (non-treated vs. cisplatin, *p* = 0.1943), log rank test. Survival was monitored for 100 days.

To evaluate whether the effects of CED of cisplatin treatment is immune-dependent, we next repeated the survival experiment in immunocompromised NSG mice. NSG mice (*n* = 6) were treated with intratumoral cisplatin at the lowest dose (0.01 μg/μl/h). The effect of cisplatin was found to be abolished without the presence of T, B and NK cells. There was no significant difference (*p* = 0.1943) in survival between non-treated NSG mice and NSG mice treated with CED of cisplatin (Fig. 3). However, we noticed a tendency towards prolonged survival in cisplatin-treated mice. Toxicity in this group reached 16.6% (1 of 6 mice) (Table 1).

### GL261 wild type-based immunotherapy does not enhance the effects of CED of cisplatin

**Figure 4:**
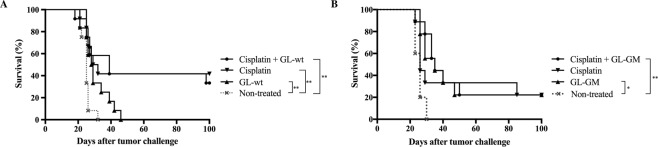
Effect of CED of cisplatin and immunotherapy on survival of GL261 tumour-bearing C57BL/6 mice. Kaplan-Meier survival curves of GL261-bearing mice receiving CED of cisplatin using a mini-osmotic pump/brain infusion kit at days 7–9, 0.01 μg/μl/h. (**A**) Addition of subcutaneous immunizations with 2 × 10^6^ irradiated GL-wt cells at days 5, 19 and 33 (*n* = 48/*n* = 12 per group). Non-treated vs. GL-wt immunization (**p = 0.0024). Non-treated vs. cisplatin (**p = 0.0025). Non-treated vs. cisplatin + GL-wt immunization (**p = 0.0050), log rank test. (**B**) Addition of intraperitoneal immunizations with 2 × 10^6^ irradiated GL-GM cells at days 5, 19 and 33 (*n* = 32/*n* = 9 for the treated groups and *n* = 5 for the non-treated group). Non-treated vs. GL-GM immunization (*p = 0.0117). Non-treated vs. cisplatin + GL-GM immunization (**p = 0.0033), log rank test. Survival was monitored for 100 days.

We have previously reported that intratumoral CED of TMZ could cure mice with intracranial GL261 tumours in an immune-dependent fashion and that immunotherapy was enhanced by this treatment^29^. Therefore, we next investigated if the lowest dose of CED of cisplatin could improve the outcome of immunizations with irradiated GL-wt cells. Immunizations were performed on days 5, 19 and 33 as described in the methods section. All non-treated mice and mice only immunized with GL-wt developed lethal tumours, however a slightly prolonged survival was observed for the immunized mice (non-treated vs. GL-wt, *p* = 0.0024) (Fig. 4A). The survival rate of mice treated with CED of cisplatin was 41.6% (5 of 12 mice), but the survival was not enhanced by addition of GL-wt immunotherapy (33.3%, 4 of 12 mice), non-treated vs. cisplatin (*p* = 0.0025), non-treated vs. cisplatin + GL-wt (*p* = 0.0050), cisplatin vs. cisplatin + GL-wt (*p* = 0.7785) (Fig. 4A).

### CED of cisplatin does not enhance the therapeutic effect of GL-GM-based immunotherapy

We have previously shown that GL-GM-based immunotherapy, e.g. immunizations with irradiated GM-CSF-secreting GL261 cells, can cure GL261-bearing mice, and that the curative effect is further enhanced by addition of either IFNγ or CED of TMZ^27–29^. We therefore next investigated whether the lowest dose of cisplatin had a beneficial effect on survival of mice immunized with GL-GM cells with the same treatment protocol as for GL-wt. 22% of GL-GM immunized mice survived tumour challenge non-treated vs. GL-GM (*p* = 0.0117) (Fig. 4B). Mice receiving intratumoral cisplatin had a 22% cure rate. The combined treatment regimen (immunization with GL-GM followed by cisplatin) had no additive effect on survival and also resulted in a 22% cure (GL-GM vs. cisplatin + GL-GM, *p* = 0.8626) (Fig. 4B).

### Immune cell infiltration following CED of cisplatin and immunotherapy

**Figure 5:**
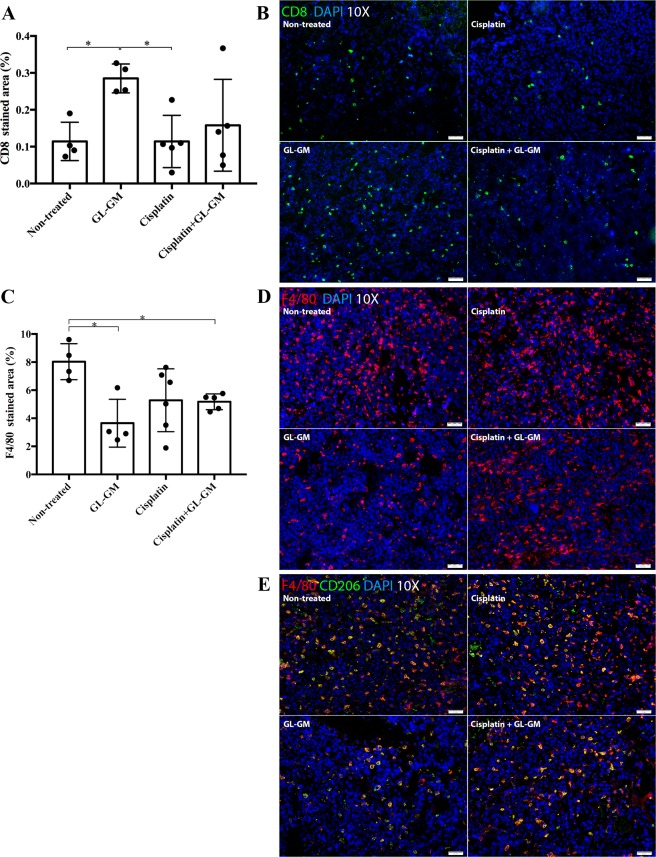
Tumour-infiltrating immune cells following CED of cisplatin and/or GL-GM immunizations. Frozen brain sections were stained for CD8^+^, F4/80^+^ and CD206^+^ cells and the percentage of stained area was determined. The median and range of each group are shown. (**A**) Tumour infiltration of CD8^+^ cells was increased in GL-GM-immunized mice compared with non-treated (*p = 0.0286) and cisplatin-treated mice (*p = 0.0159). (**B**) Representative staining of CD8 (green) in tumours. (**C**) Tumour infiltration of F4/80^+^ cells was reduced in GL-GM-immunized mice compared with non-treated mice, both as a monotherapy (non-treated vs. GL-GM, *p = 0.0286) and in combination with cisplatin (non-treated vs. cisplatin + GL-GM, *p = 0.0159). (**D**) Representative staining of F4/80 (red) in tumours. (**E**) The majority of F4/80^+^ (red) cells expressed CD206 (green). (**B**,**D**,**E**) Show representative stainings from one animal of each treatment group. Images were taken at 10x magnification. DAPI was used as a nuclear staining.

The results in NSG mice suggest that the effect of cisplatin is dependent of immune cells. Therefore, we next investigated intratumoral immune cell infiltration following cisplatin and combined treatments. Glioma-bearing mice treated with cisplatin and/or immunized with GL-GM cells were sacrificed when neurological symptoms of tumour growth appeared, the brains were snap-frozen and analysed by immunohistochemistry. Representative images of tumour sections and quantitative results of the percentage of CD8^+^ (T-cells), F4/80^+^ and CD206^+^ (macrophages) stained tumour is shown in Fig. 5. CD8^+^ and F4/80^+^ cells were detected in all treated tumours as well in non-treated tumours. However, infiltration of CD8^+^ cells was significantly higher in mice treated with immunization alone (GL-GM) compared with non-treated (*p* = 0.0286) or CED of cisplatin alone (*p* = 0.0159). The infiltration of CD8^+^ cells in tumours following treatment with cisplatin or cisplatin + GL-GM were not significantly different from non-treated tumours (Fig. 5A,B). There was no significant difference in the infiltration of CD4^+^ cells between the different treatment groups (data not shown). Furthermore, infiltration of F4/80^+^ macrophages was significantly reduced in mice treated with immunization alone (GL-GM) and the combined treatment of CED of cisplatin + GL-GM immunizations compared with non-treated. mice (*p* = 0.0286 and *p* = 0.0159, respectively) (Fig. 5C,D). A majority of F4/80+ macrophages expressed CD206 in the non-treated group as well in the treated groups (Fig. 5E).

## Discussion

In the present study, we report that cisplatin administered intratumorally using micro-osmotic pumps can cure GL261-bearing mice. The treatment effect was abolished in the absence of immune cells which has not been reported previously. However, cisplatin treatment had no additive effect in combination with either GL-wt- or GM-GM-based immunotherapy.

The GL261 cells were sensitive to cisplatin exposure *in vitro*, and also *in vivo*, as evidenced by prolonged survival compared with non-treated mice in the C57BL/6 model. The median IC_50_ *in vitro* of cisplatin was 0.81 μM which is consistent with previous *in vitro* tests in glioblastoma and other tumour cell lines^12^. This concentration is almost 40 times lower than the concentration used in the present *in vivo* experiments, which was 33 μM. The therapeutic effect of intratumorally delivered cisplatin found in these experiments is similar to previous studies in other experimental brain tumour models. The survival rate for CED of cisplatin was 39%, in contrast to survival reported by others with a range from no effect^30^ or slightly increased life span^31^ up to 13% survival^32,33^. Moreover, the total dose (0.72 μg) administered in this experiment is considerably lower than in other experiments, where the concentration was from 3–6 up to 55 μg^30–33^.

After cisplatin exposure *in vitro*, we observed a minor, however non-significant, upregulation of MHC class I expression on the GL261 cells. It has been demonstrated that cisplatin can up-regulate MHC-I in tumour cells *in vitro* and in tumour associated antigen presenting cells in a *in vivo* mesothelioma model^21^. The minor MHC-I up-regulation found in this study is probably not enough to enhance antigen presentation *in vivo*. We have also analysed MHC class I, MHC class II, CD80 and CD86 expression *in vivo* by immunofluorescent staining of cryosections (data not shown) and we could not detect any difference between tumours treated with cisplatin and immunotherapy compared to untreated tumours. The absence of upregulation of MHC class I and II, CD80 and CD86 expression on the tumour cells following cisplatin supports the notion that cisplatin does not exert its immune effect by modulation of MHC or co-stimulatory molecules on the tumour cells.

As pointed previously cisplatin is not a bona fide ICD inducer with reported release of HMGB1 and ATP but a lack of the ER stress features^26^. We could not detect any *in vivo* upregulation of surface calreticulin expression, nor any changes in other ICD related mechanistic protein such as HMGB1 or ATP in any treated group (data not shown). It has to be noted that the concept of ICD has not been explored in the setting of intratumoral delivery of cytostatic drugs. The *in vivo* testing of ICD inducers is performed by injection of cells pre-treated with the chemotherapeutic agent *in vitro*. In the present experiment cisplatin was delivered intratumorally into an established tumour and the dose adjustments might be necessary for induction of ICD.

CED has been applied in clinical therapy of brain tumours as direct intratumoral bolus injections or as infusions via catheters connected to pumps^19,34–37^. The CED route tentatively achieves higher drug concentrations and a longer exposure to the drug in the tumour while decreasing the risk of systemic toxicity^19^. CED of cisplatin administered as a single agent^38–40^ or in combination with radiotherapy^30,33^ has been reported to cure animals in several experimental glioma models. Moreover, intratumoral infusion of cisplatin has been reported feasible in 3 patients with recurrent glioblastomas but without stated survival effects^35^. There are also other feasible strategies for intratumoral drug delivery, such as carotid infusion, biodegradable polymers with slow release function^4,5,38^. Two clinical trials with intratumoral delivery of cisplatin in patients with glioblastoma have been reported with evidence of significant effect and without systemic or local toxicity^35,41^. Biodegradable polymers with cisplatin were implanted in patients after resection of primary glioblastomas with a 14.2 months overall survival compared to 7.0 months in controls^41^. In both these studies the therapy was well tolerated without any reported systemic or local toxicity. BCNU-loaded polymers showed a modest effect and were approved for treatment in primary and recurrent glioblastomas but has been mostly abandoned after the introduction of concomitant TMZ treatment^42–45^. A more recent study, using cisplatin loaded nanoparticles, could record high cure rates and less toxicity in a rat experimental glioma model^46^.

After dose reduction, we show that local neurotoxicity, as evidenced by neurological symptoms, is diminished but still present at the dose that can reject intracerebral tumours (0.01 μg/μl/h). We conclude that the therapeutic window for cisplatin is narrow as neurotoxicity was evident even in the lowest curative dose in both the C57BL6 and NSG models. The local brain toxicity of cisplatin is a general feature of platinum as local CED delivery of cisplatin in humans also entail local toxicity. The local toxicity of cisplatin can be decreased by encapsulation of the drug for slow release as evidenced by both animal studies model^39,46^ and a clinical study^41^. This indicates that local toxicity might be due to distribution of cisplatin to the brain-adjacent tumour area and normal brain. Local toxicity of cytostatic drugs most probably depends on both dose and distribution out from the tumour tissue where there is a fluid pressure in the extracellular space. To this end it has become clear that many tumours have a higher interstitial fluid pressure than the surrounding normal pressure, thus producing an additional pressure gradient^47^.

Tumour-bearing NSG mice treated with intratumoral cisplatin had a tendency towards prolonged survival compared to the controls. Since the innate and adaptive immune system in NSG is highly compromised, with depletion of T, B lymphocytes, NK cell and reduction of myeloid cell function, the results suggest that the curative effect of intratumoral cisplatin could be dependent on macrophages.

The ability of the immune system to eliminate tumour cells mostly relies on the capacity of the CD8^+^ effector cells to home to and accumulate within the tumour microenvironment. Systemic delivery of cisplatin has been reported to promote recruitment and proliferation of CD8^+^ effector cells into the tumour as well as improve their lytic activity^21^. Our results do not confirm the latter since intratumoral delivery of cisplatin did not increase CD8^+^ T cell influx while immunizations with GM-CSF-transduced cells did. In combined immunizations and cisplatin delivery there was also a tendency towards less CD8^+^ T cell influx compared to animals receiving immunization only, implying that cisplatin either reduces influx or is lethal to the T cells. Contrary to the effect of intratumoral cisplatin, intratumorally delivered TMZ had a synergistic effect when combined with GM-CSF-based immunotherapy^29^, demonstrating differences in the ability of various chemotherapeutic drugs to boost an immune response. Additionally, cisplatin has also been combined with immunotherapy preconditioning adoptive cytokine-induced killer cells by increasing the accumulation of T cells, while reducing the percentage of regulatory T cells (T-reg) intratumorally in a lung carcinoma murine model^48^.

Tumour associate macrophages recruited into the glioma environment have immune functions, and can release a wide array of growth factors and cytokines in response to those factors produced by cancer cells creating a supportive stroma for neoplastic cell expansion, survival and invasion or the opposite effect^49^. We found a significant reduction of F4/80^+^ macrophages in mice treated with immunization alone (GL-GM) and CED of cisplatin + GL-GM. In addition, we also found a reduced number of macrophages in mice treated with CED of cisplatin, however non significant. In all treatment groups, the F4/80^+^ cells were predominantly CD206^+^, suggestive of a suppressive phenotype^50,51^. Macrophages can both boost and inhibit chemotherapy by immune modulation depending on tumour type and type of chemotherapy^52^. The fact that intratumoral cisplatin does not increase neither CD8^+^ nor CD4^+^ T cell infiltration and that the effect was abolished in NSG mice (lacking T cells but with reduced macrophage function) suggests that macrophages could be partially responsible for the anti-tumour effects. Nevertheless, the effect of cisplatin may not be dependent on quantitative but rather qualitative changes in macrophages.

In conclusion, our data shows that intratumoral cisplatin by itself cured GL261-bearing mice but there was no additive effect in combination with GL-wt or GL-GM immunizations. It remains to be determined whether the absence of a synergistic effect of cisplatin combined with GL-wt- or GM-CSF-based immunotherapy was due to a non-immunogenic cell death following cisplatin treatment. The results imply that immune effects of chemotherapeutic agents are most probably dependent on the drug specificity, dosing, distribution, tumour model and type of immunotherapy. Our results cannot rule out that cisplatin can have a combined effect with other types of immunotherapies. The present results are of high relevance when designing novel clinical trials, with the aim to minimize systemic drug toxicity in the treatment of brain tumours.

By optimizing duration, time and type of local delivery of chemotherapeutic drugs in different tumour models more extensive knowledge can be achieved.

## Material and Methods

### Cell line and cell culture medium

The GL261 mouse glioma cell line of C57BL/6 origin was kindly provided by Dr. G Safrany, Hungary (GL-wt) and transduced in our lab to produce GM-CSF (GL-GM)^27^. The cells were cultured at 37 °C in the presence of 6% CO_2_ in R10-medium containing RPMI 1640 medium supplemented with 2 mM L-glutamine, 1 mM sodium pyruvate, 10 mM HEPES, 50 μg/mL gentamicin (GIBCO-Life technologies) and 10% foetal bovine serum (Biochrom AG). For tumour inoculation and immunizations, serum and gentamicin were excluded in the medium (referred to as R0-medium).

### Animals

Syngeneic female C57BL/6 mice were purchased from Scanbur (via Charles River) and male NOD-scid *IL2rγ*^*null*^ (NSG) mice were obtained from an in-house breeding core facility at BMC, Lund University. The animals were kept under specific pathogen-free conditions at BMC, Lund University. C57BL/6 mice, 8–10 weeks of age and NSG mice, 18–21 weeks, were used in the experiments. All animal procedures were performed according to the practices of the Swedish Board of Animal Research and were approved by the Committee of Animal Ethics in Lund-Malmö.

### Cisplatin and preparation of mini-osmotic pumps

The chemotherapeutic agent cisplatin 1 mg/ml (*cis*-diamminedichloroplatinum-II) (Hospira) was used for all *in vitro* and *in vivo* experiments. The cisplatin solution was dissolved in sterile 0.9% NaCl solution (Braun AG) to the appropriate concentrations: 0.9, 0.1 and 0.01 mg/ml. 3-day mini-osmotic pumps Alzet® model 1003D, 100 μl, pumping rate 1 μl/h (Nova SCB AB) were used to deliver intratumoral cisplatin. The mini-osmotic pumps were filled with 100 μl of prepared cisplatin solutions and coupled to the Alzet® brain infusion kit 3 (Nova SCB AB) with a 2.5 cm catheter tube according to the manufacturer’s protocol. The pumps assemblies were incubated at 37 °C overnight in sterile 0.9% NaCl before being placed into the mice.

### Cell viability after cisplatin exposure *in vitro*

5 × 10^4^ GL261 cells/well were seeded into a 24-well plate (Sarstedt AB). Cisplatin was diluted in R10-medium to 500, 100, 10 and 1 μM. On day 1, 2 and 3, cells were exposed to cisplatin. On day 4, the number of viable cells was assessed by 7AAD staining (BD Biosciences). The cell viability was determined by flow cytometry (Accuri®) by calculating the percentage of viable cisplatin-treated cells/viable non-treated cells. The viability of the non-treated cells was regarded as 100%. The mean and SEM values of triplicate samples were calculated.

### Measurement of MHC class I, MHC class II, CD80 and CD86 after cisplatin *in vitro*

For the evaluation of major histocompatibility complex (MHC) class I and II and co-stimulatory molecules CD80 and CD86 expression following cisplatin exposure, 5 × 10^4^ GL261 cells were exposed to 1 μM of cisplatin for 72 h. 24 h later, cells were detached using trypsin (GIBCO-Life technologies), washed in PBS (GIBCO-Life technologies) supplemented with 1% bovine serum albumin (Roche Diagnostics) and stained for 30 min at 4 °C using PE-mouse-anti-mouse-H-2Db (KH95, MHC class I), PE-mouse-anti-mouse-I-Ab (AF6-120.1, MHC class II), PE-hamster-anti-mouse-CD80 (B7-1) (16-10A1) and FITC-rat-anti-mouse CD86 (GL1) antibodies with isotype-matched controls (all from BD Biosciences Pharmingen). The expression was measured on a flow cytometer (Accuri®). The percentages of positive cells were calculated by subtraction of isotype control staining of all live cells (7AAD staining). Data was analysed using BD Accuri C6 software (Accuri®).

### Brain tumour model

On day 0 brain tumours were induced by inoculation of 5 × 10^3^ GL261-wt tumour cells in 5 μl into the right frontal lobe. Mice were placed into an induction chamber and anaesthesia was induced with 2% Isoflurane Forene® (Abbott Scandinavia AB) delivered in pure O_2_ (200 ml/s). Once the paw withdrawal reflex was absent, the mouse’s head was fixed and immobilized for the procedure in a stereotactic frame (Kopf Instruments). Isoflurane was maintained at 1.8% in spontaneous respiration. The scalp was disinfected with 70% alcohol and 0.05 ml Marcaine® (Bupivacaine hydrochloride 2.5 mg/ml + epinephrine 5 μg/ml) was injected subcutaneously. A linear skin incision starting in the midline between the eyes and ending in the midline shortly behind the bregma was performed. With a 0.5 mm rose-head a small hole was drilled into the skull 1.5 mm to the right and 1.0 mm anterior of the bregma. A Hamilton syringe (Hamilton, Switzerland) with a 33 G blunt needle was used to inject 5 μl suspension of GL261-wt cells (5 × 10^3^ cells/5 μl) 2.75 mm deep from the dural surface. The cell suspension was delivered slowly over the course of 5 min. Following injection, the needle was left in place for 3 min, then raised to a depth of 1.5 mm below the brain surface and left in place for an additional minute to diminish any back-flow through the canal. Upon withdrawal of the needle, the burr hole was sealed with bone wax and the incision was closed with one 7.5 mm metal clip.

### CED of cisplatin into immunocompetent and immunocompromised mice

On day 7, C57BL/6 (*n* = 41) and NSG (*n* = 6) tumour-bearing mice were anesthetized and fixed as described above and treated with intratumoral cisplatin. The previous skin incision was reopened, and the pump assembly pump filled with cisplatin (0.9, 0.1 and 0.01 mg/ml, delivering a total dose of 1080, 120 or 12 μg of cisplatin/kg/day for the C57BL/6 mice and 0.01 mg/ml, delivering a total dose of 12 μg of cisplatin/kg/day for the NSG mice) was implanted into a subcutaneous pocket in the midscapular area. Then, it was inserted through the premade hole in the skull and fixed to the skull using cyanoacrylate adhesive Alzet-LOCTITE® gel (Nova SCB). Finally, the incision was closed with one 7.5 mm metal clip. The pump was removed when no longer active. The survival of cisplatin-treated mice was compared with the survival of non-treated mice.

### CED of cisplatin combined with GL-wt or GL-GM immunotherapy

For survival studies of combined therapies, C57BL/6 tumour-bearing mice were randomly divided into 4 treatment groups, respectively: 1. non-treated (GL-wt *n* = 12, GL-GM *n* = 5), 2. immunization alone (GL-wt *n* = 12, GL-GM *n* = 9), 3. cisplatin (GL-wt *n* = 12, GL-GM *n* = 9) and 4. cisplatin + immunization (GL-wt *n* = 12, GL-GM *n* = 9). On day 5, 19 and 33 following tumour inoculation, mice in the immunization groups were immunized subcutaneously (GL-wt) or intraperitoneally (GL-GM) with 2 × 10^6^ irradiated (40 Grays) GL-wt or GL-GM cells in 0.2 ml R0-medium. On day 7, mice in the cisplatin groups were treated with intratumoral cisplatin as described above. The survival of cisplatin- and immunotherapy-treated mice was compared with the survival of non-treated mice.

### Endpoint in survival studies

All tumour-bearing mice were carefully observed daily for signs of drug toxicity, such as jerky or slow movement patterns and later on for neurological symptoms due to tumour growth. They were immediately euthanized when neurological symptoms appeared. All brains were examined for macroscopically visible tumours. Symptom-free surviving mice were sacrificed at the endpoint of the experiment (day 100 following tumour challenge). Cytostatic-induced toxicity has been defined as mice dying more than 3 days before the first control died. Animal that died due to toxicity were removed from the survival analysis.

### Immunohistochemistry of tumour-infiltrating immune cells

Treated tumour-bearing mice were sacrificed when neurological symptoms appeared and brains were collected, snap-frozen in liquid nitrogen-cooled isopentane (−55 °C, VWR International AB), cut into 6 μm-thick sections using a cryostat (Leica, Germany), mounted on Super frost glass slides (VWR International AB) and stored at −80 °C. Prior to staining, the sections were thawed and fixed for 10 min in 4% paraformaldehyde (VWR International AB). Sections were washed in PBS (GIBCO-Life technologies), blocked for 20 min with 5% goat serum (Jackson Immuno-Research Laboratories Inc) and stained with primary antibodies: purified rat-anti-mouse-CD8α (53–6.7) and purified rat-anti-mouse-CD4 (H129.19) (62,5 μg/ml, BD Pharmingen); rat-anti-mouse-F4/80 (CI:A3-1) (1.0 mg/ml, Bio-rad), rabbit-anti-mannose receptor (CD206) (4 μg/ml, Abcam) for 60 min at room temperature. Sections were washed, incubated with the secondary antibody for one hour, goat-anti-rat Alexa Fluor 488 IgG (2 mg/ml, Invitrogen) and donkey-anti-rat Alexa Fluor 549 IgG (2 mg/ml, Invitrogen), goat-anti-rabbit Alexa Fluor 488 IgG (2 mg/ml, Invitrogen) and mounted with Pro-long Gold anti-fading reagent containing DAPI (Molecular Probes) for nuclear staining. As negative control, primary antibodies were omitted. Images were taken at 10x and 20x magnification using a fluorescent microscope (BX-53, Olympus LRI instrument AB). Images were merged using Multi image alignment and the ratio of the stained area within the representative tumour area was calculated and expressed as percent stained area (Cellsens Dimension software, Olympus LRI instrument AB).

### Statistical analysis

Statistical differences of MHC, CD80 and CD86 expression between cisplatin-treated and non-treated cell were determined using two-way ANOVA (Fig. 1). The Kaplan-Meier survival curves were compared using a log rank test (Figs 2–4). Statistical differences between intratumoral immune cell populations were determined with non-parametric Mann Whitney *U*-test (Fig. 5). *P* values of <0.05 were considered to be statistically significant. All statistical analyses were performed using Prism7® software (GraphPad software, USA).
